# Left common iliac vein compression identified by vascular ultrasonography in asymptomatic women: does standing position influence diagnosis?

**DOI:** 10.1590/1677-5449.200188

**Published:** 2021-07-05

**Authors:** Ana Luiza Dias Valiente Engelhorn, Lucas de Brito Lima, Maria Julia Saggiorato Werka, Anna Victoria Valiente Engelhorn, Dirceu Augusto Rüdiger Bombardelli, Lucas Daniel Oliveira da Silva, Giovanna Silva Barbosa, Carlos Alberto Engelhorn

**Affiliations:** 1 Pontifícia Universidade Católica do Paraná – PUCPR, Curitiba, PR, Brasil.; 2 Faculdade Evangélica Mackenzie do Paraná – FEMPAR, Curitiba, PR, Brasil.

**Keywords:** iliac vein, compression, ultrasound

## Abstract

**Background:**

Vascular ultrasonography is the imaging exam of choice for initial screening for left common iliac vein compression, which is an asymptomatic finding that can be detected in up to 25% of some patient samples.

**Objective:**

To determine, using vascular ultrasonography, whether findings of left common iliac vein compression in asymptomatic women are different when assessed in the prone and standing positions.

**Methods:**

This is a cross-sectional observational study of 50 adult female volunteers with no symptoms of pelvic venous compression. The parameters assessed with vascular ultrasonography in the prone and standing positions were diameters and maximum velocities of the left common iliac vein at the point at which it crosses behind the right common iliac artery and before this point, in addition to left common iliac vein velocity indices at the crossing.

**Results:**

Eight cases of significant compression of the left common iliac vein were identified when assessed in prone position (16%) and just two cases (4%) were identified in the standing position. Left common iliac vein diameters were statistically larger (p = 0.002) at the point where it crosses behind the right common iliac artery in the standing position and velocities and velocity indices were statistically higher (p < 0.001) in the prone position. No significant compression of the left common iliac vein was identified in the standing position when velocity indices were normal in the prone position.

**Conclusions:**

There was no difference in detection of significant compression of the left common iliac vein when assessed in the standing position in comparison with assessment in the prone position. However, the study showed that anatomic compression of the left common iliac vein may be reduced in the standing position.

## INTRODUCTION

Compression of the left common iliac vein (LCIV) by the right common iliac artery (RCIA) is an uncommon anatomic condition that can be present in asymptomatic individuals.^1^ The LCIV crosses behind the RCIA, anteriorly from the sacral prominence of the fifth lumbar vertebra. These anatomic structures can pinch the LCIV and the combination of compression compounded by the pulsating vibration of the artery can injure the tunica intima of the vein.^2^^-^5

Compression of the LCIV by the RCIA primarily affects young and middle-aged women and is present in 22% of the population.^6^^,^^7^ This compression may be symptomatic or asymptomatic, even in cases in which the LCIV diameter is reduced by more than 70%.^8^ Complications secondary to LCIV compression are related to the risk of left iliofemoral venous thrombosis or chronic pelvic venous hypertension responsible for emergence of pelvic or lower limb varicose veins.^9^^-^12

Vascular ultrasonography (VUS) is the imaging exam of choice for initial screening for LCIV compression. Although VUS is routinely used to identify LCIV compression with patients in the prone position, there is no evidence in the literature that conducting the examination with the patient standing could yield additional useful information for diagnosis of this anatomic condition from another perspective.

The objective of this study is to use VUS to determine whether there is any difference in assessment of asymptomatic LCIV compression in women when conducted in the prone position and the standing position.

## METHODS

A cross-sectional observational study was conducted with 50 adult female volunteers. Inclusion criteria were age from 18 to 40 years and absence of symptoms of pelvic venous compression. Exclusion criteria were male sex, women over the age of 40 years, symptoms of pelvic venous compression, and lower limb varicose veins with clinical venous disease classification (CEAP) 2 to 6.

It was calculated that 46 patients were enough for this study, based on 5% level of significance, 90 % power of the test and bilateral test.

### Examination of the left common iliac vein

The women enrolled on the study were examined using Siemens^®^ Antares ultrasonography equipment (Siemens Healthcare, Issaquah, WA, USA), during the morning, after 8 h fasting, with a low frequency transducer (2 to 6 MHz). The parameters assessed in prone and standing position were: a) LCIV diameters and maximum velocities at the point where it crosses behind the RCIA; b) LCIV diameters and maximum velocities before the point at which it crosses behind the RCIA; and c) LCIV velocity index, calculated as the ratio between the maximum velocity at the point at which it crosses behind the RCIA and the maximum velocity in the LCIV segment before the crossing point.

Velocities were measured with a Doppler angle of insonation close to 60º and the diagnostic criterion adopted for significant compression was a velocity index greater than 2.5.^13^^,^^14^ Diameter and velocity variables were analyzed using Student’s *t* test for paired samples (p < 0.05) and the velocity index variable was analyzed using Wilcoxon’s nonparametric test (p < 0.05). The study was approved by the Research Ethics Committee at the Pontifícia Universidade Católica do Paraná (PUCPR), with ruling number 3.256.974.

## RESULTS

It proved technically possible to assess all the volunteers enrolled on the study, whose ages ranged from 18 to 39 years (mean of 23 years), both in the prone position and in the standing position. A total of eight cases of significant LCIV compression were identified when assessed in the prone position (16%) and just two cases (4%) were identified in the standing position. Table 1 lists the values for LCIV diameters and velocities in prone and standing positions with their respective differences.

**Table 1:** Variation in diameters and velocities in prone and standing positions.

Variable	Mean ± sd	p* (Prone x Standing)
LCIV diameter before- prone	5.03±1.46	
LCIV diameter before- standing	5.35±1.46	0.206
Difference in diam. before (Prone-Standing)	0.31±1.76	
LCIV diameter at site- prone	3.38±0.88	
LCIV diameter at site- standing	3.94±1.24	0.002
Difference in diam. at site (Prone-Standing)	0.56±1.22	
LCIV veloc. before- prone	44.21±18.99	
LCIV veloc. before-standing	26.39±10.68	< 0.001
Difference in veloc. before (Standing-Prone)	-17.82±22.34	
LCIV veloc. at site- prone	58.7±25.42	
LCIV veloc. at site- standing	33.31±13.94	< 0.001
Difference in veloc. at site (Standing-Prone)	-25.39±28.17	

* Student’s *t* test for paired samples, p < 0.05

sd = standard deviation; LCIV = left common iliac vein; diam. = diameter; veloc. = velocity.

### Diameters

**Figure 1:**
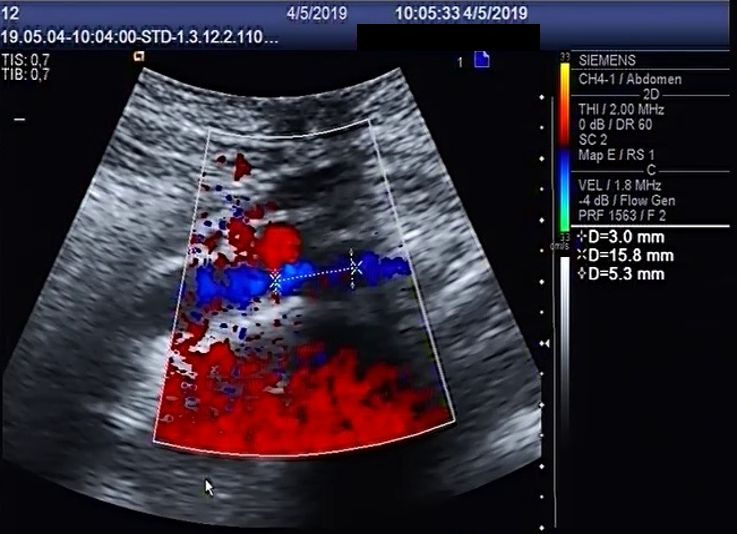
Diameter of the left common iliac vein (3 mm) at the site of its crossing behind the right common iliac artery, in the prone position.

**Figure 2:**
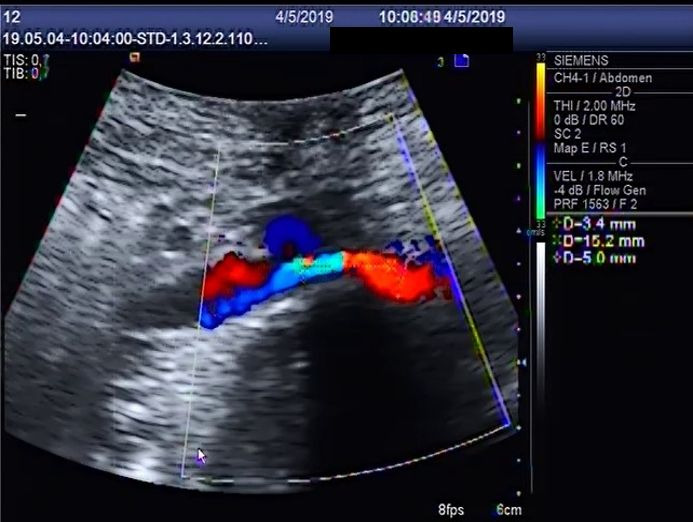
Diameter of the left common iliac vein (3.4 mm) at the site of its crossing behind the right common iliac artery, in the standing position.

The diameter of the LCIV at the point at which it crosses behind the RCIA varied from 2.9 to 6.1 mm in the prone position and from 2.2 to 7 mm in the standing position. The LCIV diameter at the point at which it crosses behind the RCIA was larger in the standing position than in the prone position (p = 0.002, Figures 1 and 2).

The LCIV diameter before the point at which it crosses behind the RCIA ranged from 2.8 to 9.4 mm in the prone position and from 2.8 to 9.5 mm in the standing position. There was no statistically significant difference (p = 0.206) in the LCIV diameter before the point at which it crosses behind the RCIA between assessments in prone position and standing position.

### Velocities

Maximum velocity in the LCIV at the point at which it crosses behind the RCIA ranged from 23 to 123 cm/s in the prone position and from 11 to 63 cm/s in the standing position. The maximum velocity in the LCIV before the point at which it crosses behind the RCIA ranged from 15 to 104 cm/s in the prone position and from 10 to 62 cm/s in the standing position.

The maximum LCIV velocity was statistically higher (p < 0.001) in prone position both at the point at which it crosses behind the RCIA and before that point.

### Velocity index

The velocity index ranged from 0.43 to 4.5 in the prone position and from 0.7 to 3.1 in the standing position. Examination in the prone position identified a higher velocity index in the LCIV (p < 0.001) than when measured in the standing position (Table 2).

**Table 2:** Variation in velocity indices in prone and standing positions

Variable	Median (minimum - maximum)	p* (Prone x Standing)
LCIV veloc. index- prone	1.54 (0.43 to 4.56)	
LCIV veloc. index- standing	1.24 (0 to 3.15)	0.080
Difference in veloc. index (Standing-Prone)	-0.30 (-3.75 to 1.3)	

* Wilcoxon’s nonparametric test, p < 0.05

veloc. = velocity; LCIV = left common iliac vein

Only two of the eight cases of LCIV compression with velocity index exceeding 2.5 found in the prone position were also identified in the standing position. No significant LCIV compression was identified in the standing position when velocity indices were normal in the prone position.

## DISCUSSION

Compression of the LCIV by the RCIA can occur in a variety of ways depending on certain anatomic elements, such as the topography of the aortic bifurcation, the topography of the junction between the LCIV and the right common iliac vein, the fifth lumbar vertebra, and possible spine curvature abnormalities (hyperlordosis). In the majority of cases (75%), the RCIA crosses over the LCIV in the territory of the junction with the right common iliac vein; in 15% of cases, this occurs a little above the junction of the common iliac veins; and in 10%, it is below the venous junction.^15^ These anatomic variations in where the artery crosses over the common iliac veins mean it is even possible for there to be compression of the right common iliac vein.^16^

Compression of the LCIV is more common in women, for whom complications secondary to compression are related to the risk of left iliofemoral venous thrombosis or chronic pelvic venous hypertension caused by the difficulty of draining venous flow, which is responsible for development of pelvic or lower limb varicose veins.^17^^-^19 After catheter fibrinolysis was introduced for treatment of iliofemoral venous thrombosis starting in the 1990s, it was observed that 50% of the patients had LCIV stenosis after venous recanalization, emphasizing the association between extrinsic LCIV compression and the risk of venous thrombosis in this segment. The reduced LCIV caliber observed in angiotomography examinations also suggests an increased risk of ipsilateral deep venous thrombosis.^20^

Since many patients have asymptomatic LCIV compression, the real incidence of the condition is unknown. In our study, we found a 16% incidence in asymptomatic women, close to estimated rates in the literature, ranging from 22 to 24%.^21^

Although VUS is routinely used to screen for LCIV compression in the prone position, there is no evidence in the literature that conducting the examination in the standing position could yield new criteria useful for diagnosis of this anatomic condition. Diagnosis of LCIV compression by the RCIA using VUS is based on anatomic identification of a reduction in the caliber of the LCIV where it crosses behind the RCIA, in addition to flow changes identified by color mapping, such as increases in velocities and flow turbulence at the site of compression, and irregularity of flow that could suggest intraluminal fibrotic bands. Indirectly, compression with possible venous obstruction can be suspected based on axial, transpelvic, or ascendant lumbar venous collateral circulation.^22^

The most relevant criterion for diagnosis of LCIV compression using VUS is the velocity index, calculated as the ratio between maximum velocity at the point at which it crosses behind the RCIA and maximum velocity at the LCIV segment before the crossing. A velocity index exceeding 2.5 suggests significant LCIV compression.^13^^,^^14^ Based on the LCIV diameters and velocity indices (> 2.5), our study of young asymptomatic women identified eight cases of significant LCIV compression when assessed in the prone position (16%) and just two cases (4%) in the standing position.

Although our study demonstrated that screening for LCIV compression in the standing position is not relevant to diagnosis with VUS, it showed that the LCIV diameters were statistically larger at the compression site in the standing position, which could indicate a relaxation of anatomic compression in this position, including reduction of maximum velocities and velocity indices — explaining the lower rate of LCIV compression and consequent lower risk of venous thrombosis in this position in relation to the prone position.

The authors conclude that there was no difference in detection of significant LCIV compression in the standing position in comparison to the prone position and that standing assessments should not be used routinely. However, the study showed that there may be reduced anatomic compression of the LCIV in the standing position, making it possible that this is a parameter to be tested when identifying more severe cases.
