# Supplementary material for: Left common iliac vein compression identified by vascular ultrasonography in asymptomatic women: does standing position influence diagnosis?
Source: J Vasc Bras. 2021 Jul 5;20:e20200188. doi: 10.1590/1677-5449.200188 (PMC8256875; doi:10.1590/1677-5449.200188)
Supplement: Translation

# Translation: Identificação pela ultrassonografia vascular da compressão da veia ilíaca comum esquerda em mulheres assintomáticas: ortostatismo pode influenciar o diagnóstico?

**Authors:** Ana Luiza Dias Valiente Engelhorn, Lucas de Brito Lima, Maria Julia Saggiorato Werka, Anna Victoria Valiente Engelhorn, Dirceu Augusto Rüdiger Bombardelli, Lucas Daniel Oliveira da Silva, Giovanna Silva Barbosa, Carlos Alberto Engelhorn

**Affiliations:** 1 Pontifícia Universidade Católica do Paraná – PUCPR, Curitiba, PR, Brasil.; 2 Faculdade Evangélica Mackenzie do Paraná – FEMPAR, Curitiba, PR, Brasil.

**Keywords:** veia ilíaca, compressão, ultrassom

## Abstract

**Contexto:**

A ultrassonografia vascular é o exame de imagem de escolha para rastreamento inicial da compressão na veia ilíaca comum esquerda, cujo achado assintomático pode ser encontrado em até 25% em algumas casuísticas.

**Objetivo:**

Identificar, pela ultrassonografia vascular, se há diferença na avaliação da compressão na veia ilíaca comum esquerda em mulheres assintomáticas em decúbito dorsal e ao ortostatismo.

**Métodos:**

Trata-se de um estudo observacional transversal em 50 mulheres voluntárias, sem sintomas de compressão venosa pélvica. Os parâmetros avaliados pela ultrassonografia vascular em decúbito dorsal e ao ortostatismo foram os diâmetros e as velocidades máximas na veia ilíaca comum esquerda no local do cruzamento com a artéria ilíaca comum direita e antes desse cruzamento, além dos índices de velocidade na veia ilíaca comum esquerda no local do cruzamento.

**Resultados:**

Foram identificados oito casos de compressão significativa na veia ilíaca comum esquerda na avaliação em decúbito dorsal (16%) e somente dois casos (4%) ao ortostatismo. Os diâmetros na veia ilíaca comum esquerda foram estatisticamente maiores (p = 0,002) no local de cruzamento com a artéria ilíaca comum direita ao ortostatismo, e as velocidades e índices de velocidades foram estatisticamente maiores (p < 0,001) em decúbito dorsal. Não houve identificação de compressão significativa na veia ilíaca comum esquerda em ortostatismo quando os índices de velocidades estavam normais em decúbito dorsal.

**Conclusão:**

Não houve diferença na detecção de compressão significativa da veia ilíaca comum esquerda ao ortostatismo em relação ao decúbito dorsal; no entanto, o estudo mostrou que pode haver menor compressão anatômica da veia ilíaca comum esquerda em posição ortostática.

## INTRODUÇÃO

A compressão da veia ilíaca comum esquerda (VICE) pela artéria ilíaca comum direita (AICD) representa uma condição anatômica pouco comum, podendo apresentar-se em indivíduos assintomáticos^1^. A VICE cruza posteriormente a AICD e localiza-se anteriormente ao promontório sacral da quinta vértebra lombar. Essas estruturas anatômicas podem causar o pinçamento da VICE, sendo a combinação da compressão somada à vibração pulsátil da artéria a causa da lesão na camada íntima do vaso^2^^-^5.

A compressão da VICE pela AICD afeta principalmente mulheres jovens e adultas de meia idade, estando presente em 22% da população^6^^,^^7^. Essa compressão pode ser sintomática ou assintomática mesmo nos casos nos quais a redução do diâmetro da VICE é superior a 70%^8^. As complicações decorrentes da compressão da VICE estão relacionadas ao risco de trombose venosa iliofemoral esquerda ou a quadros de hipertensão venosa crônica pélvica responsável pelo aparecimento de varizes pélvicas ou de membros inferiores^9^^-^12.

A ultrassonografia vascular (USV) é o exame de imagem de escolha para rastreamento inicial da compressão na VICE. Apesar de a USV ser utilizada rotineiramente para a identificação da compressão da VICE em decúbito dorsal, não existe, na literatura, evidências de que a realização do exame em ortostatismo possa adicionar novas informações úteis para o diagnóstico dessa condição anatômica, em outra perspectiva.

O objetivo deste estudo é identificar pela USV se há diferença na avaliação da compressão assintomática da VICE em mulheres em decúbito dorsal e ortostatismo.

## MÉTODOS

Foi realizado um estudo observacional transversal em 50 mulheres voluntárias. Os critérios de inclusão foram idade entre 18 anos e 40 anos e ausência de sintomas de compressão venosa pélvica. Foram excluídos os homens e as mulheres com idade superior a 40 anos ou com sintomas de compressão venosa pélvica ou de varizes dos membros inferiores com classificação clínica da doença venosa (CEAP) 2 a 6.

Foi calculado que 46 pacientes seria suficiente para este estudo, considerando-se o nível de significância de 5%, poder do teste de 90% e usando um teste bilateral.

### Avaliação da veia ilíaca comum esquerda

As mulheres incluídas no estudo foram avaliadas com equipamento de ultrassonografia Siemens^®^ Antares (Siemens Healthcare, Issaquah, WA, USA), no período da manhã, com jejum prévio de 8 h, com transdutor de baixa frequência (2 a 6 MHz). Os parâmetros avaliados em decúbito e ao ortostatismo foram: a) diâmetros e velocidades máximas na VICE no local do cruzamento com a AICD; b) diâmetros e velocidades máximas na VICE antes do cruzamento com a AICD; e c) índice de velocidade na VICE, considerando a relação entre a velocidade máxima no local do cruzamento com a AICD e a velocidade máxima no segmento da VICE antes do cruzamento.

As velocidades foram obtidas com ângulo de insonação do Doppler próximo a 60º, e o critério diagnóstico para compressão significativa foi o índice de velocidade superior a 2,5^13^^,^^14^. As variáveis diâmetros e velocidades foram avaliadas pelo teste *t* de Student para amostra pareada (p < 0,05) e a variável índice de velocidades, pelo teste não paramétrico de Wilcoxon (p < 0,05). O estudo foi aprovado pelo Comitê de Ética em Pesquisa da Pontifícia Universidade Católica do Paraná (PUCPR), sob o parecer número 3.256.974.

## RESULTADOS

Foi possível avaliar tecnicamente todas as voluntárias incluídas no estudo, cuja idade variou entre 18 e 39 anos (média 23 anos), tanto em decúbito dorsal quanto ao ortostatismo. Foram identificados oito casos de compressão significativa na VICE na avaliação em decúbito dorsal (16%) e somente dois casos (4%) ao ortostatismo. Na Tabela 1, estão detalhados os valores dos diâmetros e velocidades na VICE em decúbito e ortostatismo com suas respectivas diferenças.

**Tabela 1:** Variação dos diâmetros e velocidades em decúbito dorsal e ao ortostatismo.

Variável	Média ± desvio padrão	p* (Dec x Ort)
Diâmetro VICE pré-decúbito	5,03±1,46	
Diâmetro VICE pré-ortostatismo	5,35±1,46	0,206
Diferença Diam pré (Ort-Dec)	0,31±1,76	
Diâmetro local VICE decúbito	3,38±0,88	
Diâmetro local VICE Ort	3,94±1,24	0,002
Diferença Diam local (Ort-Dec)	0,56±1,22	
Veloc VICE pré-Dec	44,21±18,99	
Veloc VICE pré-Ort	26,39±10,68	< 0,001
Diferença Veloc pré (Ort-Dec)	-17,82±22,34	
Veloc VICE local Dec	58,7±25,42	
Veloc VICE local Ort	33,31±13,94	< 0,001
Diferença veloc local (Ort-Dec)	-25,39±28,17	

* Teste *t* de Student para amostras pareada, p < 0,05

VICE = veia ilíaca comum esquerda; Ort = ortostatismo; Dec = decúbito; Diam = diâmetro; Veloc = velocidade.

### Diâmetros

Os diâmetros da VICE no local do cruzamento com a AICD variaram entre 2,9 e 6,1 mm em decúbito e 2,2 e 7 mm ao ortostatismo. Foram identificados maiores diâmetros da VICE em ortostatismo em relação ao decúbito dorsal (p = 0,002) no local do cruzamento com a AICD (Figuras 1 e 2).

**Figura 1:** Diâmetro da veia ilíaca comum esquerda (3 mm) no local de cruzamento com a artéria ilíaca comum direita em decúbito dorsal.

**Figura 2:** Diâmetro da veia ilíaca comum esquerda (3,4 mm) no local de cruzamento com a artéria ilíaca comum direita em ortostatismo.

Os diâmetros da VICE antes do cruzamento com a AICD variaram entre 2,8 e 9,4 mm em decúbito e 2,8 e 9,5 mm ao ortostatismo. Não houve diferença estatisticamente significativa (p = 0,206) nos valores dos diâmetros da VICE em decúbito ou ortostatismo antes do local do cruzamento com a AICD.

### Velocidades

As velocidades máximas da VICE no local do cruzamento com a AICD variaram entre 23 e 123 cm/s em decúbito e 11 e 63 cm/s ao ortostatismo. As velocidades máximas da VICE antes do cruzamento com a AICD variaram entre 15 e 104 cm/s em decúbito e 10 e 62 cm/s ao ortostatismo.

As velocidades máximas na VICE foram estatisticamente maiores (p < 0,001) em decúbito dorsal tanto no local do cruzamento com a AICD quanto antes do cruzamento.

### Índices de velocidade

Os índices de velocidade vaiaram entre 0,43 e 4,5 em decúbito e 0,7 e 3,1 ao ortostatismo. Na avaliação em decúbito dorsal, foram identificados índices de velocidades maiores na VICE (p < 0,001) em relação à posição ortostática (Tabela 2).

**Tabela 2:** Variação dos índices de velocidades em decúbito dorsal e ortostatismo.

Variável	Mediana (mínimo - máximo)	p* (Dec x Ort)
Índice Veloc VICE Dec	1,54 (0,43 a 4,56)	
Índice Veloc VICE Ort	1,24 (0 a 3,15)	0,080
Diferença índice Veloc (Ort-Dec)	-0,30 (-3,75 a 1,3)	

* Teste não paramétrico de Wilcoxon, p < 0,05

Veloc = velocidade; VICE = veia ilíaca comum esquerda; Ort = ortostatismo; Dec = decúbito.

Dos oito casos de compressão na VICE com índice de velocidade superior a 2,5 em decúbito dorsal, somente dois foram identificados ao ortostatismo. Não houve identificação de compressão significativa na VICE em ortostatismo com índices de velocidades normais em decúbito dorsal.

## DISCUSSÃO

A compressão da VICE pela AICD pode ocorrer de forma variada dependendo de alguns elementos anatômicos como a topografia da bifurcação aórtica, a topografia da junção da VICE com a veia ilíaca comum direita, a quinta vértebra lombar e eventuais desvios da coluna (hiperlordose). Na maioria das vezes (75%), a AICD cruza com a VICE na topografia da junção com a veia ilíaca comum direita; em 15% dos casos, um pouco acima da junção das veias ilíacas comuns; e, em 10%, abaixo da junção venosa^15^. Essas variantes anatômicas de cruzamento arterial sobre as veias ilíacas comuns permitem a possibilidade de compressão inclusive na veia ilíaca comum direita^16^.

A compressão da VICE é mais comum em mulheres, cujas complicações decorrentes da compressão estão relacionadas a risco de trombose venosa iliofemoral esquerda ou a quadros de hipertensão venosa crônica pélvica causada pela dificuldade de escoamento do fluxo venoso responsável pelo aparecimento de varizes pélvicas ou de membros inferiores^17^^-^19. A partir da década de 1990, com o uso de fibrinolítico por cateter para tratamento de trombose venosa iliofemoral, foi observado, após a recanalização venosa, que 50% dos pacientes apresentavam estenose na VICE, reiterando a associação entre a compressão extrínseca da VICE e o risco de trombose venosa nesse segmento. A redução do calibre da VICE observada em exames de angiotomografia também sugere aumento do risco de trombose venosa profunda ipsilateral^20^.

Devido a muitos pacientes apresentarem compressão assintomática da VICE, a real incidência dessa condição é desconhecida. No nosso estudo, encontramos uma incidência de 16% em mulheres assintomáticas, taxa próxima à estimada na literatura, entre 22 e 24%^21^.

Apesar de a USV ser utilizada rotineiramente como rastreamento da compressão da VICE em decúbito dorsal, não existe, na literatura, evidência de que a realização do exame ao ortostatismo pode adicionar novos critérios úteis para o diagnóstico dessa condição anatômica. O diagnóstico da compressão da VICE pela AICD através da USV é baseado na identificação anatômica de redução do calibre da VICE no cruzamento com a AICD, além de alterações de fluxo identificadas pelo mapeamento em cores, tais como aumento de velocidades e turbilhonamento do fluxo no local de compressão, além de irregularidade do fluxo que possam sugerir bandas fibróticas intraluminais. Indiretamente, pode-se suspeitar da compressão com eventual obstrução venosa pela presença de circulação colateral venosa axial, transpélvica ou ascendente lombar^22^.

O critério mais relevante para o diagnóstico da compressão da VICE pela USV é o índice de velocidade considerando a relação entre a velocidade máxima no local do cruzamento com a AICD e a velocidade máxima no segmento da VICE antes do cruzamento. O índice de velocidade superior a 2,5 sugere compressão significativa na VICE^13^^,^^14^. Baseado nos diâmetros da VICE e nos índices de velocidades (> 2,5), nosso estudo em mulheres jovens assintomáticas identificou oito casos de compressão significativa na VICE na avaliação em decúbito dorsal (16%) e somente dois casos (4%) ao ortostatismo.

Apesar de o nosso estudo demonstrar que a pesquisa da compressão da VICE ao ortostatismo não ser relevante para o diagnóstico pela USV, mostrou que os diâmetros da VICE foram estatisticamente maiores no local da compressão ao ortostatismo, o que pode indicar um relaxamento da compressão anatômica nessa posição, inclusive com diminuição das velocidades máximas e dos índices de velocidade — o que justificaria a menor ocorrência de compressão da VICE e consequente menor risco de trombose venosa nessa posição em relação ao decúbito dorsal.

Os autores concluem que não houve diferença na detecção de compressão significativa da VICE ao ortostatismo em relação ao decúbito dorsal, e tal avaliação não deve ser realizada rotineiramente. No entanto, o estudo mostrou que pode haver menor compressão anatômica da VICE em posição ortostática, possibilitando que seja um parâmetro para ser testado na identificação de casos mais graves.
